# Decrease in serum anti‐Müllerian hormone level per puncture with laparoscopic ovarian drilling using ultrasonically activated device

**DOI:** 10.1002/rmb2.12405

**Published:** 2021-07-31

**Authors:** Shuichi Ogawa, Yusuke Atsuki, Kazuhiko Shimada, Mitsuhiro Motoyama, Tatsuya Suzuki, Hiroyuki Fujiwara

**Affiliations:** ^1^ Institution of Central Clinic Shimotsuke‐shi Japan; ^2^ Department of Obstetrics & Gynecology Jichi Medical University Shimotsuke‐shi Japan

**Keywords:** anti‐Müllerian hormone (AMH), body mass index (BMI), laparoscopic ovarian drilling (LOD), polycystic ovary syndrome (PCOS), ultrasonically activated device

## Abstract

**Purpose:**

To determine the contributing factor in infertility treatment with laparoscopic ovarian drilling (LOD) to the decrease in serum anti‐Müllerian hormone (AMH) levels in patients with polycystic ovarian syndrome using an ultrasonically activated device.

**Methods:**

A retrospective analysis was performed in 60 patients (aged 23–36 years) who received 25–120 punctures in each ovary with LOD treatment from January 2014 to December 2018.

**Results:**

The mean decrease in serum AMH level per puncture with LOD was 0.07 ± 0.04 ng/ml in all 60 patients and 0.08 ± 0.04 ng/ml in patients with ≥10 ng/ml preoperative serum AMH level, which was significantly higher than in those with <10 ng/ml (0.05 ± 0.02 ng/ml). The mean decrease in serum AMH level per puncture in patients with body mass index (BMI) < 18.5 kg/m^2^ (0.10 ± 0.03 ng/ml) was significantly higher than in those with BMI 18.5–25 kg/m^2^ (0.07 ± 0.04 ng/ml) and >25 kg/m^2^ (0.06 ± 0.02 ng/ml).

**Conclusions:**

The mean decrease in serum AMH levels per puncture with LOD using an ultrasonically activated device depends on the preoperative serum AMH level and BMI of patients.

## 1 INTRODUCTION

Laparoscopic ovarian drilling (LOD) has been widely used to treat polycystic ovary syndrome (PCOS).^1^, ^2^, ^3^, ^4^, ^5^, ^6^ In general, the clinical results indicate that LOD restores menstrual cyclicity and ovulation in approximately 70–80% of patients, and pregnancy rate following LOD is approximately 40–60%.^2^, ^3^, ^6^, ^7^ There was no statistically significant difference in pregnancy or miscarriage rates between LOD and gonadotropin therapy.^3^, ^7^ LOD is performed through various methods: electrocautery; lasers including CO_2_, argon, KTP, and Nd‐YAG; and ultrasonically activated devices.^2^, ^8^, ^9^, ^10^ In LOD using electrocautery, power setting, duration of penetration, depth of penetration, and the number of punctures to be applied vary in each case because it is impossible to objectively measure the cauterized area of the ovarian cortex.^1^, ^2^, ^3^, ^5^, ^6^ In addition, LOD with lasers, laser machines, power settings, and the number of punctures were different.^7^, ^8^, ^10^, ^11^ LOD using electrocautery or laser is influenced by the subjectivity and experience of the operator.

Serum anti‐Müllerian hormone (AMH) is useful in diagnosing ovarian function, and LOD is expected to normalize the high preoperative serum AMH levels in patients with PCOS. A decrease in serum AMH level after LOD compared to the preoperative value appears to be a good predictor of the ovarian response to LOD^12^; however, this has not been clarified yet.

In LOD using an ultrasonically activated device, the effect of treatment can be assessed only by the number of applied punctures.^9^

In this study, we retrospectively determined the decrease in serum AMH level per puncture with LOD in 60 patients with PCOS who received LOD treatment at our clinic. The ultimate goal of this study was to establish a formula for the number of punctures with LOD required to achieve the target serum AMH level. However, the decrease in serum AMH level per puncture would depend on patient background. Therefore, we conducted an exploratory analysis of potential confounding factors such as preoperative serum AMH levels.

## 2 MATERIALS AND METHODS

### 2.1 Subjects

A retrospective analysis was performed in 60 patients (aged 23–36 years) with PCOS who received 25–120 punctures in each ovary with LOD treatment from January 2014 to December 2018 at the Institution of Central Clinic. LOD was indicated in patients who met any of the following criteria: (I) had PCOS with clomiphene citrate (CC) and/or letrozole resistance and no desire for gonadotropin therapy; (II) had PCOS with failed infertility treatment with CC and/or letrozole and no desire for gonadotropin therapy; and (III) had PCOS with failed infertility treatment with CC and/or letrozole at high risk of ovarian hyperstimulation syndrome (OHSS) by gonadotropin therapy and no desire for in vitro fertilization, and had desired for LOD in any case.

PCOS was diagnosed when at least two of the following three criteria were satisfied, as proposed by the Rotterdam Consensus Meeting^13^: oligomenorrhea or amenorrhea, clinical hyperandrogenism and/or hyperandrogenemia, and polycystic ovaries.

### 2.2 Laparoscopic ovarian drilling

**FIGURE 1:**
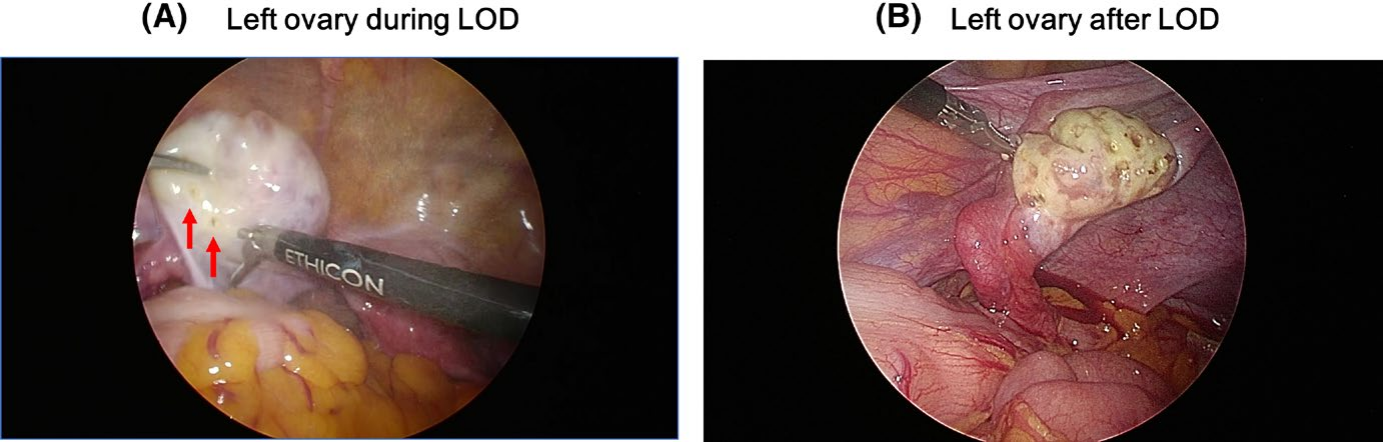
Ovary during and after laparoscopic ovarian drilling (LOD). (A) Left ovary during LOD. Harmonic Scalpel II drilling the ovary to obtain 2‐mm^2^ punctures. **↑** Red arrows indicate holes drilled through the ovarian capsule by an ultrasonically activated device. (B) Left ovary after LOD. A total of 100 punctures were made. During surgery, bleeding could occur, but it will stop spontaneously after the procedure [Colour figure can be viewed at wileyonlinelibrary.com]

The LOD was performed under general anesthesia using a three‐puncture laparoscopy method. A 5‐mm laparoscope was inserted via the sub‐umbilical route, and two 5‐mm manipulating trocars were introduced through the right and left lower abdominal incisions. An ultrasonically activated device (Harmonic Scalpel II [curve shears with 5 mm diameter and 36 cm shaft length]: Ethicon Endo‐Surgery, Johnson & Johnson K.K. Japan) was used to penetrate the ovarian capsule. The ovary was held with a grasper, and a laparoscopic forceps blade was inserted from the contralateral side (Figure 1A). The tip of the active blade was placed perpendicularly onto the ovarian surface, and 25–120 punctures per ovary were applied with the highest power setting level of 5. Internal fluid was drained after the blade reached the antral follicles properly. Fifty to 220 punctures were performed in total (Figure 1B) according to the operator's subjectivity based on preoperative serum AMH level and ovary size. It takes approximately 2–3 s to make a single puncture; therefore, the total duration of drilling was <10 min, and the total operation time was approximately 30–40 min.

### 2.3 Follow‐up data

The serum AMH level was determined using the original protocol of the AMH electrochemiluminescence immunoassay (SRL Inc., Japan). Serum AMH level was measured within 2 months before and 1 month after LOD to retrospectively calculate the decrease in serum AMH level per puncture using the following formula:

$$\text{The decrease in serum AMH level per puncture}=\left(\text{preoperative serum AMH level}‐\text{postoperative serum AMH level}\right)/\text{total number of punctures}$$

The results were also categorized by two age groups: <32 years and ≥32 years, based on a study reporting that the fecundity of women decreases significantly around 32 years of age and decreases more rapidly after 37 years of age,^14^ by body mass index (BMI): <18.5 kg/m^2^ (underweight), 18.5–25 kg/m^2^ (normal weight), or >25 kg/m^2^ (overweight and obesity) according to the BMI classification by WHO,^15^ and by preoperative serum AMH level <10 ng/ml or ≥10 ng/ml based on a study reporting that >97% of women with highly elevated AMH levels (>10 ng/ml) had PCOS.^16^

All data were analyzed using the IBM Statistical Package for Social Sciences for Windows (version 11.0; Armonk, NY, USA). Welch's *t*‐test was used to compare the results. Statistical significance was set at *p* < .05.

## 3 RESULTS

### 3.1 Baseline characteristics of patients

The baseline characteristics of the 60 patients enrolled in the study are presented in Table 1. The mean age (± *SD*) was 29.8 ± 3.2 years, and the mean BMI (± *SD*) was 23.0 ± 4.8 kg/m^2^. All patients were diagnosed with PCOS according to the Rotterdam criteria and underwent LOD. Of the 60 patients, 20 were classified into PCOS criterion I, 19 in criterion II, and 21 in criterion III.

**TABLE 1:** Baseline characteristics of the study participants

Characteristic	Mean ± *SD*	*n* (%)
Age (years)		
Overall	29.8 ± 3.2	60
32–36		16 (26.7)
23–31		44 (73.3)
Infertility type		
Primary		45 (75)
Secondary		15 (25)
Cycle history		
Amenorrhea		5 (8.3)
Oligomenorrhea		55 (91.7)
Regular		0 (0)
Body mass index (kg/m^2^)		
Overall	23.0 ± 4.8	60
<18.5		7 (11.7)
18.5–25		38 (63.3)
25–30		8 (13.3)
>30		7 (11.7)
Anti‐Müllerian hormone (ng/ml)		
Overall	12.7 ± 6.5	60
<10		23 (38.3)
≥10		37 (61.7)

### 3.2 Natural ovulatory rate and pregnancy rate after LOD

The natural ovulatory rate after LOD was 76.7% (46/60) in total, with 95% (19/20) in criterion I, 57.9% (11/19) in criterion II, and 76.2% (16/21) in criterion III. The mean postoperative AMH levels in the group with and without natural ovulation after LOD were 3.79 ± 2.56 ng/ml and 4.47 ± 1.85 ng/ml, respectively. No significant difference was observed between the two groups (*p* = .276).

Thirty‐one patients (51.7%) became pregnant, and 28 (46.7%) delivered healthy infants. Of the 31 pregnant patients, 13 achieved pregnancy in natural ovulation cycles, 12 in CC cycles, 1 in letrozole cycle, 3 in CC‐human menopausal gonadotropin (HMG) cycles, and 2 in recombinant follicular stimulation hormone (FSH) cycles. In our clinic, fertility treatment is performed at natural ovulation for the patients who received LOD, and in cases where natural ovulation does not occur, CC is administered. Of the 12 pregnant patients who received CC after LOD, 3 were CC‐sensitive and 9 were CC‐resistant before starting their treatment. They accounted for 25% (5/20) of patients in the PCOS criterion I, 15.8% (3/19) in II, and 19% (4/21) in III, respectively.

Of the 60 patients who received LOD treatment, 10 received gonadotropin therapy, 3 received CC‐HMG therapy, and 7 received recombinant FSH therapy. Two patients were judged to have high risk of OHSS during ovulation induction, and the treatments were discontinued, but the other eight patients proceeded to HCG treatment. Based on the OHSS severity rating by Brinsden et al.,^17^ mild OHSS was observed in 37.5% (3/8) of the patients who received HCG.

BMI‐stratified analysis showed that the pregnancy rates after LOD were 57.1% (4/7) in patients with BMI < 18.5 kg/m^2^, 47.3% (18/38) in those with BMI 18.5–25 kg/m^2^, and 60% (9/15) in those with BMI > 25 kg/m^2^. No certain trend related to BMI was found.

### 3.3 Decrease in serum AMH level after LOD

**FIGURE 2:**
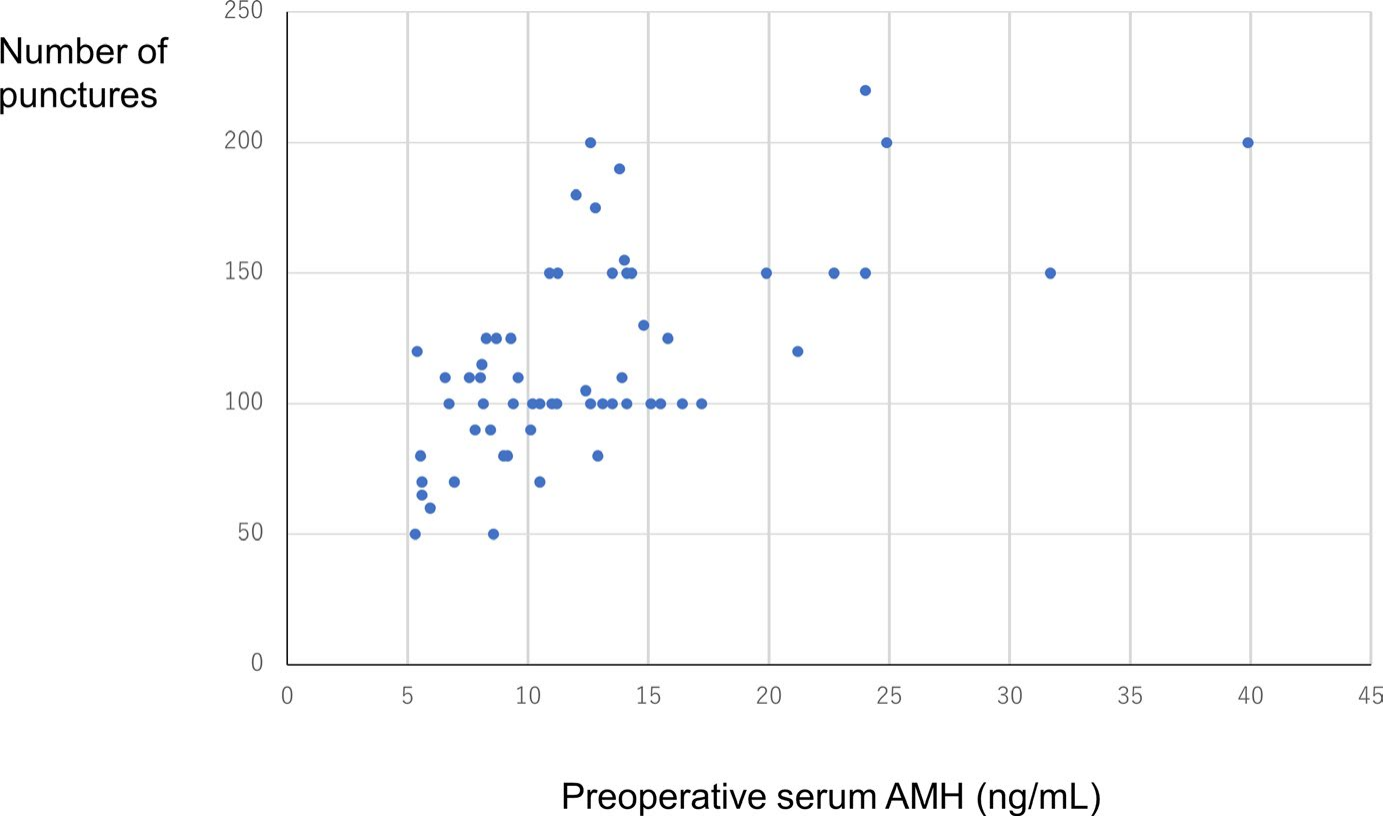
Number of ovarian punctures with laparoscopic ovarian drilling by preoperative serum anti‐Müllerian hormone (AMH) level (ng/ml) [Colour figure can be viewed at wileyonlinelibrary.com]

Serum AMH levels were measured within 2 months before and 1 month after the LOD. The number of ovarian punctures with LOD based on preoperative serum AMH levels is shown in Figure 2. One hundred and more punctures were performed in 91.9% (34/37) and 52.3% (12/23) of patients with preoperative serum AMH levels ≥10 ng/ml and <10 ng/ml, respectively. One hundred and fifty or more punctures were performed in 45.9% (17/37) of patients with preoperative serum AMH levels ≥10 ng/ml because their ovaries were 1.5‐ to 2‐fold larger than normal. None of the patients with preoperative serum AMH level <10 ng/ml had ovaries 1.5‐fold larger than normal. The mean (± *SD*) preoperative and postoperative serum AMH levels were 12.70 ± 6.50 and 3.96 ± 2.41 ng/ml, respectively. The mean (± *SD*) decrease in serum AMH level per puncture with LOD was 0.07 ± 0.04 ng/ml (range: 0.01–0.19) in all 60 patients.

**FIGURE 3:**
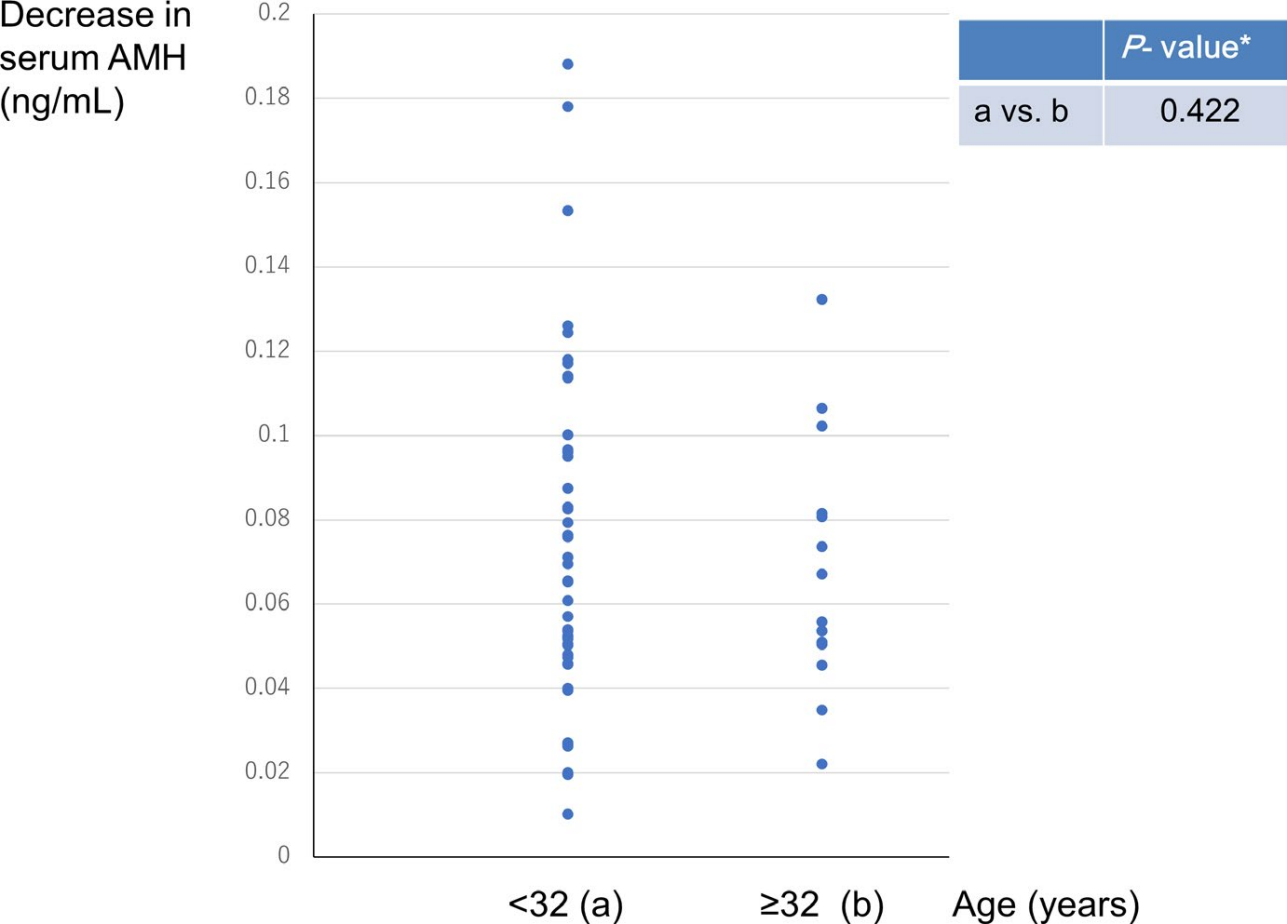
Decrease in serum anti‐Müllerian hormone (AMH) level per puncture (ng/ml) with laparoscopic ovarian drilling (LOD) by age category (years) at the time of LOD. [Mean ± *SD*] age < 32: 0.07 ± 0.04 ng/ml, age ≥ 32: 0.07 ± 0.03 ng/ml. [Median] age < 32: 0.07 ng/ml, age ≥ 32: 0.05 ng/ml. *Welch's *t*‐test [Colour figure can be viewed at wileyonlinelibrary.com]

Age‐stratified analysis showed that the mean (± *SD*) preoperative and postoperative serum AMH levels were 12.20 ± 3.01 ng/ml and 4.21 ± 2.94 ng/ml, respectively, in patients aged ≥32 years, and 12.88 ± 7.40 ng/ml and 3.87 ± 2.22 ng/ml in those aged <32 years. There was no significant difference in the mean (± *SD*) decrease in serum AMH level per puncture with LOD between patients aged ≥32 years (0.07 ± 0.03 ng/ml) and those aged <32 years (0.07 ± 0.04 ng/ml, *p* = .422) (Figure 3).

**FIGURE 4:**
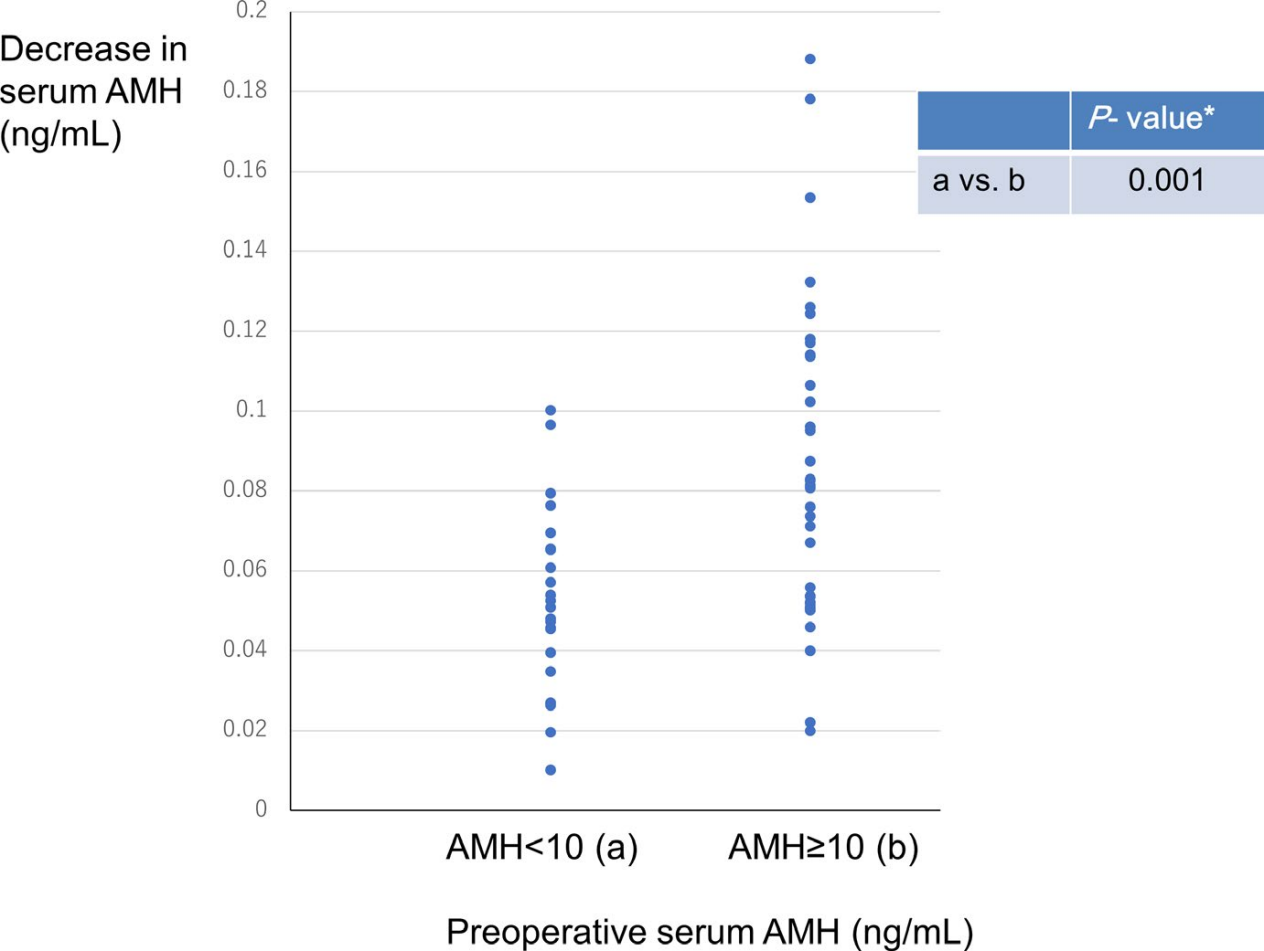
Decrease in serum anti‐Müllerian hormone (AMH) level per puncture (ng/ml) with laparoscopic ovarian drilling by preoperative serum AMH level (ng/ml). [Mean ± *SD*] preoperative serum AMH ≥ 10 ng/ml: 0.08 ± 0.04 ng/ml, preoperative serum AMH < 10: 0.05 ± 0.02 ng/ml. [Median] preoperative serum AMH ≥ 10 ng/ml: 0.08 ng/ml, preoperative serum AMH < 10 ng/ml: 0.05 ng/ml. *Welch's *t*‐test [Colour figure can be viewed at wileyonlinelibrary.com]

On comparing preoperative serum AMH levels, the mean (± *SD*) postoperative serum AMH level was 4.70 ± 2.62 ng/ml in patients with ≥10 ng/ml of preoperative serum AMH level (mean preoperative value: 15.90 ± 6.36 ng/ml) and 2.76 ± 1.39 ng/ml in those with <10 ng/ml of preoperative serum AMH level (mean preoperative value: 7.55 ± 1.44 ng/ml). The mean decrease in serum AMH level per puncture in patients with ≥10 ng/ml of preoperative serum AMH level (0.08 ± 0.04 ng/ml) was significantly higher than in those with <10 ng/ml of preoperative serum AMH level (0.05 ± 0.02 ng/ml, *p* = .001) (Figure 4).

**FIGURE 5:**
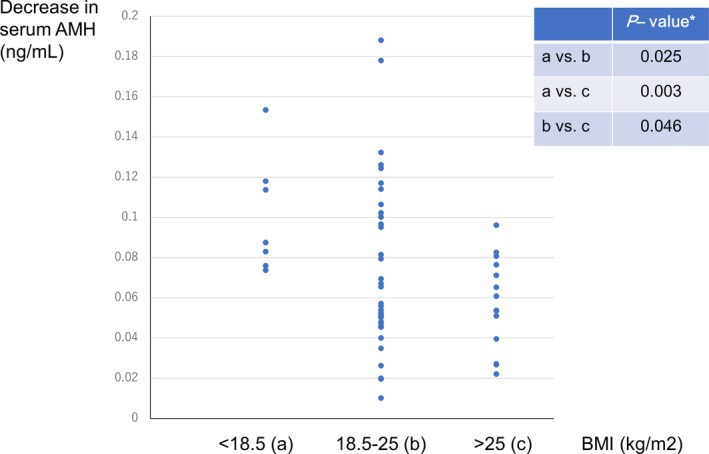
Decrease in serum anti‐Müllerian hormone (AMH) level per puncture (ng/ml) with laparoscopic ovarian drilling by body mass index range (BMI, kg/m^2^). [Mean ± *SD*] BMI < 18.5 kg/m^2^: 0.10 ± 0.03 ng/ml, 18.5 kg/m^2^ ≤ BMI ≤ 25 kg/m^2^: 0.07 ± 0.04 ng/ml, BMI > 25 kg/m^2^: 0.06 ± 0.02 ng/ml. [Median] BMI < 18.5 kg/m^2^: 0.09 ng/ml, 18.5 kg/m^2^ ≤ BMI ≤ 25 kg/m^2^: 0.05 ng/ml, BMI > 25 kg/m^2^: 0.05 ng/ml. *Welch's *t*‐test [Colour figure can be viewed at wileyonlinelibrary.com]

The BMI‐stratified analysis showed that the mean (± *SD*) preoperative and postoperative serum AMH levels were 15.19 ± 5.04 ng/ml and 4.20 ± 2.29 ng/ml, respectively, in patients with BMI < 18.5 kg/m^2^; 12.62 ± 7.14 ng/ml and 3.65 ± 2.22 ng/ml, respectively, in those with BMI 18.5–25 kg/m^2^; and 11.74 ± 5.32 ng/ml and 4.63 ± 2.89 ng/ml, respectively, in those with BMI > 25 kg/m^2^. The mean (± *SD*) decrease in serum AMH level per puncture in patients with BMI < 18.5 kg/m^2^ (0.10 ± 0.03 ng/ml) was significantly higher than in those with BMI 18.5–25 kg/m^2^ (0.07 ± 0.04 ng/ml, *p* = .025) and >25 kg/m^2^ (0.06 ± 0.02 ng/ml, *p* = .003) (Figure 5). The mean decrease in serum AMH level per puncture in patients with BMI 18.5–25 kg/m^2^ was significantly higher than in those with BMI > 25 kg/m^2^ (*p* = .046) (Figure 5).

## 4 DISCUSSION

In 1935, Stein and Leventhal reported seven cases of PCO, where wedge resection of the ovaries during laparotomy was first employed, resulting in normal ovulation and pregnancy.^18^ The procedure has been effective for patients with CC‐resistant PCOS, but there is a risk of postoperative adhesion formation leading to mechanical infertility. Laparoscopic ovarian electrocautery and ovarian drilling for the treatment of PCOS have been reported since the 1980s.^1^, ^2^, ^3^, ^4^, ^5^, ^6^ It is now recognized that LOD is an effective second‐line treatment for anovulatory and oligo‐ovulatory infertility associated with PCOS.

In the LOD using an ultrasonically activated device, a blade is placed onto the surface of the ovarian capsule to induce a shock wave called cavitation, which breaks tissues.^9^ Bleeding is rare, and there is no excess heat generation in the blade; therefore, damage to the ovary can be minimized. Unlike electrocautery, the ultrasonically activated device does not cauterize the ovarian tissues surrounding the puncture site.

It is expected that LOD treatment can eliminate excess small follicles located directly beneath the ovarian capsule and facilitate the development of new healthy follicles. The only setting on the device to be made is puncture area and depth at 2 mm^2^ and up to 10 mm, respectively. The effect of the LOD treatment seems to depend solely on the number of punctures to be applied.

According to a study by Dumont et al., FSH stimulates the conversion of testosterone to estradiol in pre‐antral follicles, and AMH inhibits the action of FSH. In women with PCOS, underdeveloped pre‐antral follicles and lack of ovulation leads to an increase in AMH levels, which directly inhibits the action of FSH and reduces the secretion of estradiol. Therefore, a higher AMH level is more resistant to ovulation induction by ovarian stimulation.^19^

The effects of LOD were objectively evaluated by analyzing ovulation, serum LH/FSH ratio, number of antral follicles, and free androgen index.^20^ Recently, serum AMH levels have been recognized as a more objective index for the assessment of LOD treatment.^12^ Postoperative reduction mechanism in AMH levels could involve the atresia of several small follicles as part of normal follicular development leading to ovulation. However, there seems to be no consensus regarding the appropriate timing to measure postoperative AMH levels in assessing the clinical efficacy of LOD. Therefore, this study utilized data from postoperative checkups conducted 1 month after LOD.

We started LOD treatment in 2010 according to Aoki's report stating that the appropriate number of punctures with LOD is approximately 40–50 per ovary.^21^ However, no clinical efficacy was observed in patients with large ovaries (1.5‐fold larger than normal), requiring a second LOD. Therefore, we increased the number of punctures per ovary to approximately 100 in patients with large ovaries, after which clinical efficacy was observed in patients even with a single LOD. Therefore, we decided to perform LOD with 25–120 punctures per ovary in this study.

The biggest concern in treating PCOS patients with LOD is determining the exact number of punctures required to impart maximum clinical effect while preventing substantial decrease in ovarian functions caused by LOD. Therefore, we explored the possibility of serum AMH levels before LOD as an index for deciding the number of punctures.

The target AMH level decrease after LOD was set at 1.0–2.0 ng/ml plus age‐specific mean AMH values.^22^ Although LOD is a recognized method to lower AMH levels in PCOS patients, a formula for calculating the adequate number of punctures has not been established. Therefore, it would be clinically useful to predict serum AMH levels after LOD using a simple index.

In this study, we found that the mean decrease in serum AMH level per puncture applied with LOD was 0.07 ± 0.04 ng/ml in 60 patients, and it was significantly larger in patients with preoperative serum AMH level ≥10 ng/ml than in those with preoperative serum AMH level <10 ng/ml. Bhide et al. reported that the median AMH/antral follicle count ratio in PCOS patients, asymptomatic polycystic ovarian morphology (PCOM), and control groups were 1.92, 1.13, and 1.00, respectively, and sub‐fertile patients with PCOS secreted significantly more AMH per antral follicle than patients with PCOM only and control patients.^23^ The mean decrease in serum AMH level was highly correlated with preoperative serum AMH level in this study, which could be related to basal AMH production.

Meanwhile, it is unclear why the mean decrease in serum AMH level is larger in patients with BMI < 18.5 kg/m^2^. There have been no reports of premature ovarian failure after LOD^24^, ^25^; however, it is necessary to pay attention to excess LOD for patients with BMI < 18.5 kg/m^2^.

Based on the data obtained from this study, we determined that 0.07 ng/ml was the standard value of the decrease in serum AMH level per puncture with LOD using an ultrasonically activated device. The decrease in serum AMH level per puncture with LOD in the PCOS patients with BMI < 18.5 kg/m^2^ was larger and that in those with BMI > 25 kg/m^2^ was lower than the standard value of 0.07 ng/ml. Therefore, we determined that a decrease in serum AMH level per puncture with LOD in patients with BMI < 18.5 kg/m^2^ was 0.07–0.1 ng/ml and that in those with BMI > 25 kg/m^2^ was 0.05–0.07 ng/ml considering the preoperative BMI.

We conducted an analysis with preoperative serum AMH levels, BMI, and age as confounding factors. The results of the *t*‐test for each factor showed that the decrease in serum AMH level per puncture was correlated with preoperative serum AMH levels and BMI.

The natural ovulatory rate of the 60 patients investigated was 76.7% (46/60) after LOD. Thirty‐one patients (51.7%) became pregnant, and 28 patients (46.7%) delivered healthy infants after LOD. The clinical effects observed in this study seem to be similar to those in previous reports.^2^, ^3^, ^4^, ^7^, ^8^, ^9^, ^10^, ^11^

However, this study has several limitations. It should be noted that the results and discussions presented in this study are based on exploratory research and not confirmatory clinical trials. The ultimate goal of this study was to establish a predictive formula for calculating the number of punctures with LOD required to reach the proper target level of serum AMH after LOD. To achieve this goal, a multivariate regression model is indispensable, and further data need to be collected to reach a conclusion, but we have not reached a clear conclusion to make a predictive hypothesis to determine the number of punctures in LOD. In addition, it is difficult to predict pregnancy outcomes based on the decrease in serum AMH levels in the study with such a small sample size, considering other factors for the establishment of pregnancy. In fact, we had cases where pregnancies after LOD were achieved not only by coitus but also by intrauterine sperm insemination. However, this clinical finding suggests that LOD contributes to the improvement of fertility. Hendriks et al. (2014) also showed that LOD contributes to a decrease in testosterone, androstenedione, AMH, and insulin growth factor‐1.^11^ Considering these facts, it is suggested that a study with a large sample is necessary to verify the results of this exploratory study.

In conclusion, 0.07 ng/ml is the standard value of the decrease in serum AMH level per puncture with LOD using an ultrasonically activated device in patients with PCOS, and it fluctuates depending on preoperative serum AMH level and BMI. We expect that LOD will be more widely recognized as one of the options for reducing AMH levels to accelerate the accumulation of data to clarify the correlation between the number of punctures and AMH levels and between AMH reduction and fertility improvement and will further contribute to the improvement of fertility in patients with PCOS.

## CONFLICTS OF INTEREST

The authors have no conflicts of interest to declare.

## HUMAN RIGHTS STATEMENTS AND INFORMED CONSENT

All the procedures accorded with the ethical standards of the relevant committees on human experimentation (institutional and national) and with the Helsinki Declaration of 1964 and its later amendments. The study design was approved by the ethics committee of Institutional Review Board of Institute of Central Clinic. This is a retrospective study in patients who submitted informed consent for undergoing fertility treatment at our clinic.

## ANIMAL STUDIES

This article does not contain any study with animal participants that have been performed by any of the authors.
